# Development and psychometric validation of the affective evaluation towards pubertal changes scale for late childhood (9–12 years old) Indonesian children

**DOI:** 10.3389/fpsyg.2026.1793400

**Published:** 2026-05-29

**Authors:** Indri Utami Sumaryanti, Fitri Ariyanti Abidin, Annemarie Samuels, Aulia Iskandarsyah

**Affiliations:** 1 Faculty of Psychology, Universitas Padjadjaran, Sumedang, Indonesia; 2 Faculty of Psychology, Bandung Islamic University, Bandung, Indonesia; 3 Department of General, Develpomental, and Educational Psychology, Faculty of Psychology, Universitas Padjadjaran, Sumedang, Indonesia; 4 Center for Relationship, Family Life and Parenting Studies, Faculty of Psychology, Universitas Padjadjaran, Sumedang, Indonesia; 5 Institute of Cultural Anthropology and Development Sociology, Leiden University, Leiden, Netherlands; 6 Department of Clinical and Health Psychology, Faculty of Psychology, Universitas Padjadjaran, Sumedang, Indonesia

**Keywords:** affective evaluation towards pubertal changes, Indonesian children, late childhood, psychometric validation, scale development

## Abstract

**Introduction:**

Puberty represents a major developmental transition involving several aspects. In Indonesia, limited open discussion about puberty, and the widespread use of non- standardized instruments have constrained efforts to assess children’s psychological readiness for pubertal changes. This study aimed to develop and validate a developmentally appropriate, culturally relevant Affective Evaluation toward Pubertal changes Scale for boys and girls aged 9–12 years.

**Methods:**

A quantitative instrument development design was employed, including item generation, expert-based content validation, pilot testing, Exploratory Factor Analysis (EFA) using a polychoric matrix with unweighted least squares extraction and oblique rotation, and that the number of factors determined by parallel analysis. Confirmatory Factor Analysis (CFA) was conducted and the WLSMV estimator used for ordinal data. A bifactor model also tested to verify the presence of a general attitude factor. Calculating omega hierarchical and explained common variance. Discriminant validity assessed via HTMT and invariance between boys and girls tested through multigroup CFA. Data were collected from 514 elementary school children and randomly divided into independent samples for EFA (*n* = 264) and CFA (*n* = 250).

**Results:**

This study evaluated the factorial structure, construct validity, reliability, and gender- based measurement invariance of a newly developed instrument. EFA supported a three-factor structure. CFA demonstrated good model fit across multiple indices. Initial assessment indicated marginal convergent validity for one factor; however, model respecification improved Average Variance Extracted values. Discriminant validity was generally acceptable. Bifactor indices suggested a predominantly unidimensional structure with meaningful specific factors. Reliability estimates indicated high internal consistency for each factor and the overall scale. Multi-Group CFA employed across gender, although a relatively high HTMT value between two factors was observed in the male subgroup.

**Discussion:**

The findings provide strong preliminary support for the factorial validity, reliability, and cross-gender applicability of the instrument. The three-factor structure was empirically supported through both exploratory and confirmatory analyses, and dimensionality assessment indicated the presence of a dominant general factor alongside distinct subdimensions. Measurement invariance across gender suggests that the instrument operates equivalently for male and female respondents. However, the elevated discriminant validity estimate between two factors in the male group suggests further investigation.

## 1 Introduction

Puberty represents a critical developmental transition marked by rapid biological, psychological, and social changes. While pubertal development is biologically driven, children’s psychological readiness to understand, accept, and cope with these changes does not necessarily develop in parallel with physical maturation. Research indicates that early pubertal changes, when not accompanied by adequate psychological readiness, may lead to confusion, fear, anxiety, and negative attitudes toward bodily changes (Carskadon and Acebo, 1993; Petersen et al., 1988). These psychological responses are particularly salient during early adolescence, a developmental period in which children begin to form foundational beliefs about their bodies and self-identity.

Globally, assessment of puberty has largely focused on physical maturation through instruments such as the Pubertal Development Scale, which evaluates secondary sexual characteristics but does not capture children’s emotional or attitudinal preparedness for puberty-related changes (Carskadon and Acebo, 1993). In contrast, psychological constructs such as puberty readiness and attitudes toward puberty reflect children’s cognitive understanding, emotional responses, and behavioral preparedness for bodily changes. These constructs have been shown to play an important role in adolescent adjustment, influencing mental health outcomes, body image, and coping behaviors (Graber et al., 2018).

Cultural context plays a crucial role in shaping how children perceive and respond to pubertal changes. Most widely used puberty-related psychological instruments, including the Menstrual Attitude Questionnaire, were developed in Western sociocultural contexts characterized by relatively open communication about bodily development (Brooks-Gunn and Ruble, 1980). Cross-cultural research suggests that attitudes toward puberty and menstruation are strongly influenced by cultural beliefs and social norms. Attitudes toward puberty are socially and culturally constructed; therefore, valid measurement requires instruments that are sensitive to local beliefs, family messages, and social norms shaping children’s interpretations of pubertal changes (Liu et al., 2012; Weichold et al., 2025). In many Indonesian communities, discussions about puberty are often considered sensitive or taboo, which may limit children’s access to accurate information and emotional support during early pubertal transitions.

In Indonesia, empirical studies on puberty-related readiness have predominantly focused on girls and menarche, particularly among elementary and junior high school students. Several studies have documented that many Indonesian girls experience insufficient readiness to face menarche, often characterized by fear, anxiety, and limited understanding of bodily changes (Lutfiya, 2017; Mardalena, 2018; Rumiyandini et al., 2021). Psychological factors such as knowledge, attitudes, and attachment to caregivers have been found to be significantly associated with readiness to face menarche (Hartini, 2015; Hidayah and Palila, 2018; Perangin-angin and Kembaren, 2021). Intervention studies further suggest that health education can improve readiness, indicating that psychological preparedness is modifiable and measurable (Novita, 2020).

Despite the growing body of Indonesian research, most studies rely on researcher- developed questionnaires with varying conceptual definitions and limited psychometric validation. The widespread use of non-standardized instruments constrains comparability across studies and limits the generalizability of findings at the national level (Solehah, 2018). Moreover, existing Indonesian instruments are largely gender-specific and focus almost exclusively on menarche, leaving boys’ experiences of puberty underexplored. This is concerning, as international evidence indicates that boys also experience confusion, emotional distress, and uncertainty during early pubertal changes, particularly when lacking appropriate guidance (Graber et al., 2018).

Psychological readiness for puberty has important implications for children’s mental health and well-being. Research suggests that adolescents with insufficient knowledge and readiness regarding pubertal changes are more likely to employ avoidance-oriented coping and experience heightened emotional stress (Farid et al., 2019). Indonesian studies similarly report that inadequate readiness to face puberty-related changes may negatively affect children’s emotional responses and adaptation, highlighting the need for early assessment and preventive intervention (Lutfiya, 2017; Mardalena, 2018).

Early adolescence, particularly the age range of 9–12 years, represents a sensitive developmental period during which children begin to experience or anticipate pubertal changes. Instruments designed for older adolescents may be developmentally inappropriate for this age group due to complex language or assumptions of prior pubertal experience. Currently, Indonesia lacks a standardized, age-appropriate, and gender-inclusive instrument to assess affective evaluation towards pubertal changes among children in late childhood.

Given the increasing occurrence of early pubertal onset, the cultural specificity of puberty-related experiences, the predominance of non-standardized and gender-limited measurement approaches in Indonesian research, and the critical importance of early adolescence as a formative developmental stage, there is a clear need to develop and validate a scale tailored to Indonesian children aged 9–12 years that measure affective evaluation towards pubertal changes. Such a scale would support higher-quality research, facilitate evaluation of school-based puberty and health education programs, and enable early psychological interventions aimed at promoting healthy developmental trajectories among Indonesian children.

## 2 Materials and methods

### 2.1 Study design

This study employed a quantitative methodological design aimed at developing and validating a psychological instrument to measure attitudes toward puberty among Indonesian children aged 9–12 years. The instrument development process followed established guidelines for scale construction in psychology, including item generation, content validation, pilot testing, and statistical analysis.

#### 2.1.1 Phase 1: conceptualization and item generation

**Figure 1:**
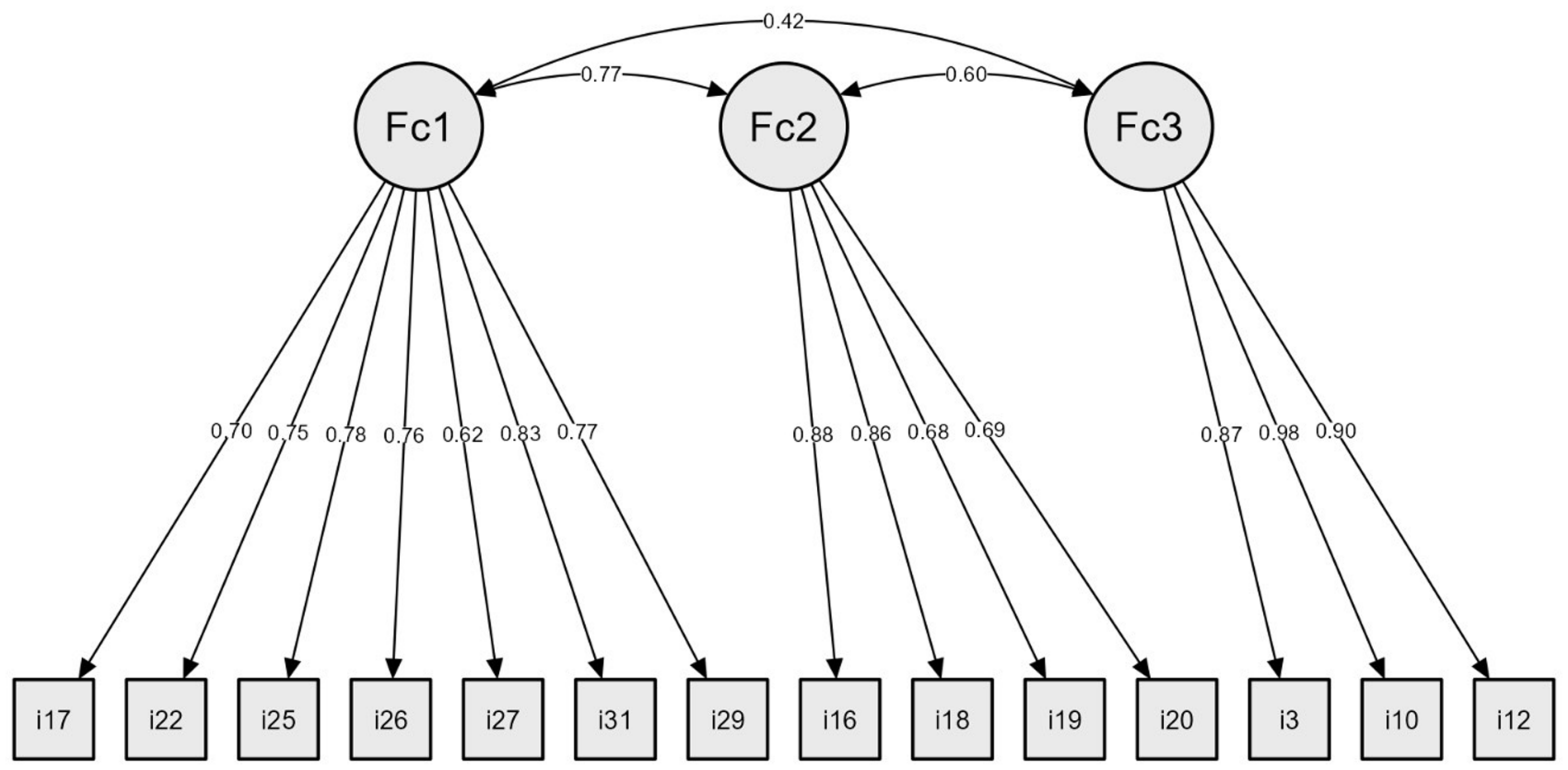
Confirmatory factor analysis respecification.

The construct of affective evaluation towards pubertal changes was conceptualized as a multidimensional aspect. Prior to the development of the Scale a pilot implementation of a school-based sexuality education program conducted in four schools. The program was designed and implemented by the researchers, who were authors of this paper. The school- based intervention delivered to students included information on physical pubertal changes based on endocrine changes that experienced by both boys and girls, neurobiology changes regarding thinking and emotional aspect, as well as psychological and social changes grounded in Erikson’s psychosocial development theory. During the implementation of the program, students expressed various reactions, comments, and questions, which were systematically documented and compiled by the research team (see Figure 1).

##### 2.1.1.1 Theoretical definitions of physical/endocrine pubertal changes

Endocrine theory provides a coherent framework for explaining the physical manifestations of puberty. Development of axillary hair and contribute to the onset of adult- type body odor by activating apocrine sweat glands, the onset of adult-type body odor (Rosenfield, 2021), oily skin and contributes to the pathogenesis of acne vulgaris (Del Rosso and Kircik, 2024), pubertal growth spurt (Styne, 2003). Collectively, these findings demonstrate that acne, body odor, axillary hair growth, oily skin, and rapid somatic growth are not isolated phenomena but integrated outcomes of endocrine maturation during adolescence.

##### 2.1.1.2 Operational definition of affective evaluation towards physical/endocrine pubertal changes

Physical pubertal changes are defined as the affective evaluation (like-dislike) towards somatic developments during adolescence that result from activation of the hypothalamic pituitary gonadal (HPG) axis and increased adrenal androgen production. For the purposes of this study, physical pubertal changes will be operationalized as the presence and/or degree of the following endocrine-driven indicators: axillary hair development, onset of adult-type body odor, oily skin, acne vulgaris, pubertal growth spurt.

##### 2.1.1.3 Theoretical definition of neurobiological pubertal changes

Puberty is associated not only with physical changes but also with profound neurobiological maturation that underlies emotional and cognitive development. Implicated in emotion and prefrontal regions responsible for higher-order cognition (Casey et al., 2008; Arain et al., 2013). Thus, neurobiological evidence indicates that puberty plays a significant role in shaping both emotional richness and abstract cognitive capacity, as the adolescent brain increasingly integrates hormonal influences with ongoing neural maturation to support complex thinking and emotional processing.

##### 2.1.1.4 Operational definition of neurobiological pubertal changes

Neurobiological pubertal changes are defined as affective evaluation (like-dislike) towards alterations in brain structure, brain function, and hormonally influenced cognitive- emotional capacities that occur during adolescence as a result of pubertal hormonal activation and ongoing neural maturation.

For the purposes of this study, neurobiological pubertal changes will be operationalized using the following indicators: Emotional Processing Maturation (emotional intensity, emotional regulation, or affective variability), Prefrontal Cognitive Development (abstract reasoning developed).

##### 2.1.1.5 Theoretical definition of psychosocial pubertal changes

Puberty exerts a significant psychosocial impact, particularly within the stage of Identity versus Role Confusion (Erikson, 1994). Puberty increased self-awareness, intensifying this developmental task by heightening sensitivity to peer evaluation, body image, and social comparison, thereby amplifying identity exploration processes (Kroger, 2017).

##### 2.1.1.6 Operational definition of psychosocial pubertal changes

Psychosocial pubertal changes are defined as affective evaluation (like-dislike) towards increases in self-awareness, identity exploration, and sensitivity to peer evaluation that emerge during adolescence as puberty intensifies the developmental task of identity formation.

For the purposes of this study, psychosocial pubertal changes will be operationalized through the following indicators: Identity Exploration and Commitment, Self-Awareness, Peer Evaluation Sensitivity and Body Image Concern.

Drawing upon both the theoretical framework and the practical observations from the intervention, an initial pool of 31 items was generated under the construct affective evaluation toward pubertal changes. This construct measures the affective evaluation of pubertal changes consisting of: physical (endocrine and neurobiological) changes, psychological changes and social changes. Written in Bahasa Indonesia using a 5 points Likert scale ranging from 1 (Dislike) to 5 (Really like), to ensure comprehension among children.

#### 2.1.2 Phase 2: content validity

Content validity was assessed using expert judgment. A panel of 3 experts including developmental psychologists, educational psychologists and elementary school educators evaluated each item for relevance, clarity, cultural appropriateness, and age suitability.

Experts rated item relevance using a 4-point scale. The Content Validity Index (CVI) was calculated at both the item level (I-CVI) and scale level (S-CVI). Items with an I- CVI below 0.78 were revised or removed. Feedback from experts was also used to simplify wording and ensure cultural sensitivity. The CVI procedures followed established guidelines for content validity assessment (Lynn, 1986; Polit and Beck, 2006; Polit et al., 2007).

#### 2.1.3 Phase 3: pilot testing

A pilot study was conducted with 12 children aged 9–12 years recruited from elementary schools in Bandung. The objectives of the pilot testing were to assess item clarity and comprehension, response patterns, completion time and preliminary internal consistency. Children were encouraged to ask questions during administration, and problematic items were identified through observation and item analysis. Based on pilot results, ambiguous or poorly performing items were revised or eliminated.

#### 2.1.4 Phase 4: main study participants and data collection

##### 2.1.4.1 Participants

Participants were children in late childhood aged 9–12 years (boys and girls) enrolled in public and private elementary schools in Bandung City and Urban region. Inclusion criteria were aged between 9 and 12 years, ability to read and understand Bahasa Indonesia, parental consent and child assent. Children with diagnosed developmental or cognitive impairments that could interfere with questionnaire comprehension were excluded.

##### 2.1.4.2 Sampling procedure and sample size

A stratified purposive sampling strategy was employed. Schools were first categorized based on school type (public district, public municipal, private Islamic, and private non- religious) and geographical location. Invitations were then distributed to schools within each category, and those that agreed to participate were included in the study. Permission to conduct the study in public schools was obtained from the local education authority, while approval for private schools was granted by the respective school principals. This approach ensured representation across key institutional and regional characteristics while acknowledging the non-probability nature of the sampling procedure.

For factor analysis, sample size recommendations of 5–10 participants per item were followed. A total of *N* = 514 participants were recruited and randomly divided into two independent samples. Sample 1 (EFA): *n* = 264. Sample 2 (CFA): *n* = 250 with boys *n* = 111, girls *n* = 139.

To avoid overfitting and to ensure replication of the factor structure, exploratory factor analysis (EFA) and confirmatory factor analysis (CFA) were conducted on two independent samples. The first sample was used for EFA, and the second sample, comprising different participants, was used for CFA.

##### 2.1.4.3 Procedure

Data collection was conducted in classroom settings under researcher supervision. Parental informed consent and child assent were obtained prior to participation. Questionnaires were administered collectively, and researchers provided standardized instructions. Completion time ranged from approximately 15–20 min.

##### 2.1.4.4 Statistical analysis

This was an initial stage analysis. To analyze the first set of data, Exploratory Factor Analysis will be conducted using a polychoric matrix with unweighted least squares extraction and oblique rotation, and that the number of factors will be determined by parallel analysis. To analyse the second set of data, Confirmatory Factor Analysis was conducted and the WLSMV estimator used for ordinal data. A bifactor model also tested to verify the presence of a general attitude factor (Rodriguez et al., 2016), calculating omega hierarchical and explained common variance. Discriminant validity assessed via HTMT (Roemer et al., 2021), and invariance between boys and girls tested through multigroup CFA (configural, metric, and scalar).

## 3 Results

### 3.1 Statistical analysis

#### 3.1.1 Exploratory factor analysis

Exploratory Factor Analysis (EFA) was conducted using a polychoric correlation matrix with promax rotation to identify the underlying factor structure of the research instrument. Sampling adequacy was supported by a Kaiser–Meyer–Olkin (KMO) value of 0.832, indicating meritorious sampling adequacy, and a significant Bartlett’s Test of Sphericity (*χ*^2^ = 2670.474; df = 465; *p* < 0.001), confirming that the correlation matrix was suitable for factor analysis.

The initial extraction yielded seven factors based on the eigenvalue-greater-than-one criterion. However, results from parallel analysis and examination of the scree plot indicated that a three-factor solution provided the most optimal structure. Therefore, subsequent analyses were performed by specifying a three-factor model. Items with factor loadings below 0.40 and/or high uniqueness values (> 0.70) were eliminated to enhance construct clarity and factor stability (Costello and Osborne, 2005). Specifically, items 1, 2, 4, 5, 6, 7, 8, 9, 11, 13, 14, 15, 24, 28, and 30 (15 items) were removed because they did not demonstrate adequate contribution to the emerging latent factor structure.

Following item refinement, the final three-factor model demonstrated improved psychometric properties. All retained items exhibited factor loadings ≥ 0.40. The factor loading values ranged from 0.418 to 0.858 for Factor 1, 0.630 to 0.951 for Factor 2, and 0.718 to 0.841 for Factor 3. These results indicate that each retained indicator contributed meaningfully to the representation of its respective latent construct.

Collectively, the three factors accounted for a total variance of 0.530, equivalent to 53.0% of the total variance in the data. This finding suggests that more than half of the variability in respondents’ responses can be explained by the derived factor structure, indicating adequate representational capacity of the model. In social science and psychometric research contexts, a total explained variance exceeding 50% is generally considered indicative of a sufficiently strong and stable factor structure suitable for subsequent analyses.

#### 3.1.2 Confirmatory factor analysis

Model fit evaluation was conducted using both absolute and comparative fit indices based on the WLSMV estimator to validate the proposed three-factor structure. The results indicated that the model demonstrated good fit to the data, with *χ*^2^(101) = 141.146, *p* = 0.005, CFI = 0.976, TLI = 0.971, RMSEA = 0.040 [90% CI (0.023, 0.055)], and SRMR = 0.040. These values suggest that the three-factor model adequately represents the observed data (CFI and TLI ≥ 0.95; RMSEA ≤ 0.06; SRMR ≤ 0.08; Hu and Bentler, 1999).

Examination of the standardized factor loadings indicated satisfactory psychometric characteristics of the retained indicators. Several indicators demonstrated moderate loadings (> 0.50), while approximately half of the indicators exhibited strong loadings (> 0.70). These findings support the adequacy of the measurement model in representing the intended latent constructs.

Convergent validity was assessed using the Average Variance Extracted (AVE). Factors F2 and F3 yielded AVE values of 0.525 and 0.554, respectively, exceeding the recommended threshold of 0.50 and indicating good convergent validity (Fornell and Larcker, 1981), meaning that each construct explains more than half of the variance of its indicators. In contrast, Factor F1 produced an AVE value of 0.465, which falls slightly below the recommended criterion, suggesting that further refinement or review of the items associated with F1 may be warranted.

Discriminant validity was subsequently evaluated using the Heterotrait–Monotrait ratio (HTMT). HTMT values ranged from 0.399 to 0.813, with the highest value observed between Factors F1 and F2 (HTMT = 0.813). The remaining relationships were moderate (F2– F3 = 0.609) to low (F1–F3 = 0.399). All HTMT values were below the conservative threshold of 0.85, indicating adequate discriminant validity and suggesting that the constructs are empirically distinct from one another (Henseler et al., 2015). Although the association between F1 and F2 was relatively higher compared to other factor pairs, it remained within acceptable limits and does not pose concerns regarding discriminant validity.

#### 3.1.3 Dimensionality and reliability

To evaluate construct dimensionality, bifactor indices were also examined. The omega hierarchical coefficient (*ω*h = 0.70) indicates that approximately 68% of the reliable variance in the total score is attributable to the general factor, suggesting a relatively strong though not exclusively dominant general factor. In addition, the Explained Common Variance (ECV = 0.60) demonstrates that the general factor accounts for 60% of the shared variance among items (Rodriguez et al., 2016). Taken together, these findings support the presence of a substantial general factor while also indicating that the specific factors continue to provide meaningful contributions. Accordingly, the scale can be characterized as predominantly unidimensional with relevant multidimensional components.

Finally, reliability analysis demonstrated high internal consistency. McDonald’s omega coefficients ranged from 0.786 to 0.886, while Cronbach’s alpha values ranged from 0.789 to 0.883 across factors. The overall scale reliability reached ω = 0.920 and *α* = 0.902. These results confirm that the instrument possesses excellent reliability and consistently measures the intended construct (Nunnally and Bernstein, 1994).

#### 3.1.4 Model respecification

Following the initial Confirmatory Factor Analysis (CFA), the measurement model indicated that several indicators did not fully satisfy the criteria for convergent validity, as reflected by the Average Variance Extracted (AVE) for Factor 1 (0.465), which fell below the recommended minimum threshold of 0.50. Therefore, a model respecification was undertaken to refine the measurement structure based on the evaluation of parameter estimates. The respecification process involved reviewing factor loadings, examining each indicator’s contribution to its respective construct, and assessing the overall impact on model quality. Based on this evaluation, items 21 and 23 from Factor 1 were removed due to their relatively low contribution, as indicated by smaller factor loadings compared to the remaining indicators. Item deletion was performed incrementally while ensuring that the conceptual integrity of the construct was preserved.

After respecification, discriminant validity was reassessed using the Heterotrait– Monotrait ratio (HTMT), yielding values ranging from 0.406 to 0.788, all below the conservative threshold of 0.85. The highest HTMT value was observed between Factor 1 and Factor 2 (HTMT = 0.788), demonstrating a reduction compared to the previous model. The HTMT values for Factor 2–Factor 3 and Factor 1–Factor 3 were 0.609 and 0.406, respectively. These results indicate that the factors remain empirically distinguishable. Furthermore, AVE values for all factors after respecification met the ≥ 0.50 criterion, with AVE values of 0.502 for Factor 1, 0.525 for Factor 2, and 0.554 for Factor 3. This indicates that each factor explains more than half of the variance in its respective indicators. Consequently, the respecified measurement model satisfies both convergent and discriminant validity criteria and is deemed suitable as the final model for subsequent analyses, including the assessment of measurement invariance across groups.

#### 3.1.5 Measurement invariance across gender

After the respecification measurement model was deemed to meet construct validity and reliability criteria, the next step involved conducting Multi-Group Confirmatory Factor Analysis (MGCFA) to examine measurement invariance between male and female groups. Invariance testing was performed sequentially across three levels: configural invariance, metric invariance, and scalar invariance. Model evaluation was based on multiple fit indices, including the Comparative Fit Index (CFI), Tucker–Lewis Index (TLI), Root Mean Square Error of Approximation (RMSEA), and Standardized Root Mean Square Residual (SRMR) (Hu and Bentler, 1999).

At the configural level, the model demonstrated acceptable fit, with CFI = 0.943, TLI = 0.930, RMSEA = 0.069 [90% CI (0.052, 0.085)], and SRMR = 0.058. These results indicate that the factorial structure was equivalent across groups, suggesting that the pattern of relationships between items and latent factors was similarly configured for males and females. The chi- square test yielded *χ*^2^ = 236.393 with df = 148 and *p* < 0.001, indicating that the proposed factor model provided a significantly better fit than the baseline model. Therefore, the configural model was considered appropriate as the foundation for subsequent invariance testing.

Parameter estimates showed that all standardized factor loadings exceeded the minimum threshold of 0.50 in both groups. In the female group, loadings ranged from 0.565 to 1.081, whereas in the male group, loadings ranged from 0.520 to 0.847, with all estimates statistically significant (p < 0.001). These findings suggest that each indicator contributed adequately to its respective latent construct in both groups.

Discriminant validity assessed using the Heterotrait–Monotrait ratio (HTMT) in the configural model revealed values ranging from 0.320 to 0.681 in the female group and from 0.479 to 0.910 in the male group. Most values were below the conservative threshold of 0.85, supporting adequate discriminant validity. However, in the male group, the HTMT value between Factor 1 and Factor 2 was 0.910, slightly exceeding the conservative cutoff, indicating a relatively strong association between these two factors. Although this warrants cautious interpretation, overall discriminant validity remained acceptable.

Regarding convergent validity, Average Variance Extracted (AVE) values in the configural model showed that, in the female group, AVE was 0.485 for Factor 1, 0.597 for Factor 2, and 0.657 for Factor 3, with Factors 2 and 3 meeting the ≥ 0.50 criterion. In the male group, AVE for Factor 1 was 0.504, meeting the recommended threshold, whereas Factor 2 (0.466) and Factor 3 (0.476) were slightly below the suggested cutoff.

At the metric invariance level, model fit indices showed minimal changes relative to the configural model, with CFI = 0.940, TLI = 0.931, RMSEA = 0.069 [90% CI (0.052, 0.084)], and SRMR = 0.068. The chi-square value was χ^2^ = 252.057 with df = 159 and *p* < 0.001. The small change in fit indices suggests that constraining factor loadings to be equal across groups did not substantially reduce model fit, indicating that the strength of the relationships between items and factors was equivalent for males and females. Factor loadings remained above 0.50 and statistically significant (*p* < 0.001) in both groups, confirming that the instrument measured the constructs in a comparable manner across gender.

HTMT values at the metric level displayed a similar pattern to the configural model, ranging from 0.361 to 0.681 in the female group and from 0.491 to 0.910 in the male group. The majority of values remained below the 0.85 threshold, although the association between Factor 1 and Factor 2 in the male group remained relatively high. This suggests conceptual proximity between certain constructs, but overall discriminant validity was still maintained.

AVE values at the metric level indicated that, in the female group, AVE was 0.492 for Factor 1, 0.595 for Factor 2, and 0.657 for Factor 3. In the male group, AVE for Factor 1 was 0.503 (meeting the criterion), while Factor 2 (0.473) and Factor 3 (0.477) remained slightly below 0.50. Overall, convergent validity remained acceptable.

At the scalar invariance level, the model demonstrated CFI = 0.932, TLI = 0.928, RMSEA = 0.070 [90% CI (0.055, 0.085)], and SRMR = 0.071. Although a slight decrease in model fit was observed, all indices remained within the acceptable range. The chi-square test yielded *χ*^2^ = 274.554 with df = 170 and *p* < 0.001. These results indicate that item intercepts can be considered equivalent across groups, supporting meaningful comparison of latent means between males and females.

HTMT values at the scalar level remained consistent with previous stages, ranging from 0.361 to 0.681 in the female group and from 0.491 to 0.910 in the male group. Most values remained below recommended thresholds, suggesting that discriminant validity was generally preserved despite the relatively high association between Factor 1 and Factor 2 in the male group.

AVE values at the scalar level showed that, in the female group, AVE was 0.487 for Factor 1, 0.595 for Factor 2, and 0.656 for Factor 3. In the male group, AVE for Factor 1 was 0.501 (meeting the criterion), while Factor 2 (0.472) and Factor 3 (0.477) remained slightly below the recommended threshold. Nonetheless, the overall model quality remained acceptable.

In summary, changes in model fit indices from configural to metric and scalar levels were relatively small and remained within recommended limits. These findings indicate that the measurement model demonstrates satisfactory invariance across gender groups. Therefore, the model achieves scalar invariance and is suitable for comparing latent constructs between males and females.

## 4 Discussion

### 4.1 Alignment with developmental and cultural context

Consistent with prior research highlighted in the introduction, the present study confirms that children in late childhood (9–12 years) already hold distinct, structured affective evaluation toward pubertal changes that extend beyond physical maturation alone. This conclusion is strongly supported by the factor-analytic evidence. Sampling adequacy and factorability were clearly established (KMO = 0.832; Bartlett’s Test of Sphericity *χ*^2^ = 2670.474, df = 465, *p* < 0.001), indicating that the inter-item correlation pattern was appropriate for identifying latent constructs.

Although the initial extraction yielded seven factors under the eigenvalue-greater-than- one rule, parallel analysis and the scree plot converged on a three-factor solution as the most conceptually and statistically defensible structure. This three-factor configuration reflecting social, physical, and psychological dimensions fits well with developmental models positioning puberty as a multidimensional transition involving bodily change, emotional meaning-making, and shifting social roles (Dorn and Biro, 2011). The emergence of a clear social factor in late childhood is therefore developmentally coherent. As children approach adolescence, they increasingly integrate physical changes into their social identities, a process shaped by peer norms and cultural expectations (Mendle et al., 2007). In contexts where open discussion of puberty may be limited as is often reported in more conservative or modesty- oriented cultures children may rely more heavily on peer discourse and implicit social cues to interpret pubertal transitions (Deardorff et al., 2007). From an ecological perspective, children’s interpretations of biological changes are embedded within microsystem influences such as family communication patterns and peer interactions (Bronfenbrenner and Morris, 2006). Thus, in the Indonesian cultural context, limited formal dialogue about puberty may amplify the role of socially constructed meanings in shaping affective evaluations.

### 4.2 Psychosocial dimension

A major conceptual contribution of this study is the identification of a distinct psychosocial dimension, capturing children’s internal meaning-making about puberty (e.g., feelings of maturity, independence, and transition into adolescence). The existence of this factor supports the introduction’s emphasis that psychological readiness is not an automatic byproduct of biological development. Statistically, this is reinforced by the observed separation between physical and psychosocial factors: the data indicate that awareness of bodily changes does not necessarily translate into emotional acceptance or positive identity integration.

The retained indicators demonstrated meaningful contributions to their constructs, with strong loading patterns across factors after refinement. Specifically, the final EFA solution showed loadings ranging 0.418–0.858 (Factor 1), 0.630–0.951 (Factor 2), and 0.718–0.841 (Factor 3), indicating that children’s responses were not random or diffuse but organized around coherent latent dimensions (Hair et al., 2019). The presence of moderate-to-strong loadings across retained items implies that these affective evaluations towards physical, psycho-social changes are already “formed enough” to be measured reliably before adolescence fully unfolds.

From an applied perspective, these findings directly support the implication raised in the introduction: puberty education should not be limited to biological facts. Because psychosocial interpretation forms a distinct domain, interventions should include emotional coping, identity framing, and normalization of mixed feelings, rather than assuming that increased knowledge alone will resolve anxiety or fear. This finding is developmentally meaningful, as late childhood is increasingly recognized as a preparatory phase for identity- related processes that intensify in adolescence (Steinberg, 2014).

### 4.3 Measurement contributions and psychometric strength

From a measurement standpoint, the study directly addresses the literature gap identified in the introduction namely, the frequent use in Indonesian puberty research of non- standardized, gender-specific, or insufficiently validated tools. The instrument development process (item development → expert validation → pilot testing → psychometric evaluation) resulted in a scale with strong empirical support at multiple levels.

#### 4.3.1 EFA refinement and factor stability

To strengthen construct clarity, items with loadings below 0.40 and/or high uniqueness (> 0.70) were removed, resulting in the elimination of 15 items (1, 2, 4, 5, 6, 7, 8, 9, 11, 13, 14, 15, 24, 28, 30). This step is psychometrically important: it indicates the final construct representation is not inflated by weak indicators and improves interpretability and replicability.

#### 4.3.2 Explained variance

The three factors accounted for 53.0% of total variance. This level is typically considered adequate to strong for attitudinal constructs in social science contexts, especially with younger respondents whose self-report responses can be more heterogeneous. Notably, this also clarifies an inconsistency with the earlier narrative that variance explained was “modest”: empirically, the total explained variance exceeds 50%, supporting a substantively meaningful representation of the construct space.

#### 4.3.3 CFA model fit and measurement quality

The three-factor structure was further supported using WLSMV estimation with good fit indices: *χ*^2^(101) = 141.146, *p* = 0.005; CFI = 0.976, TLI = 0.971, RMSEA = 0.040 [90% CI (0.023, 0.055)], SRMR = 0.040. This pattern indicates that the proposed structure reproduces observed covariance relationships well, with error levels consistent with a well-specified measurement model.

#### 4.3.4 Convergent and discriminant validity

Convergent validity via AVE initially showed strong performance for Factors 2 and 3 (AVE = 0.525 and 0.554), while Factor 1 was slightly below the recommended cutoff (AVE = 0.465). Discriminant validity was supported through HTMT values ranging from 0.399 to 0.813, all below the conservative 0.85 threshold, with the highest association between F1 and F2 (HTMT = 0.813) suggesting conceptual proximity but not overlap severe enough to threaten distinctness.

#### 4.3.5 Bifactor evidence and interpretation

Bifactor indices indicated a meaningful general factor (*ω*h = 0.70; ECV = 0.60), suggesting that approximately 60% of the shared variance among items is attributable to a broad overarching construct, while the specific factors still retain meaningful unique variance. Substantively, this aligns with how puberty readiness may operate: children may hold an overall readiness/attitude orientation, yet also differentiate their experiences into social, physical, and psychological subdomains. Therefore, the instrument can be interpreted as predominantly unidimensional with important multidimensional components, supporting flexible use (total score and/or subscale scores) depending on research or program evaluation needs.

#### 4.3.6 Reliability

Internal consistency was excellent: McDonald’s ω ranged from 0.786 to 0.886 across factors, Cronbach’s *α* from 0.789 to 0.883, and the overall reliability was *ω* = 0.920 and *α* = 0.902. These values strongly indicate that the scale provides stable measurement for both research and applied screening/evaluation purposes.

### 4.4 Implications for research and practice

The results reinforce the argument advanced in the introduction that late childhood is a critical window for assessment and preventive intervention. Because the factor structure is already coherent in ages 9–12 and supported by strong loadings, variance explained (53%), and excellent reliability, the instrument enables earlier identification of dislike evaluation towards pubertal changes that may increase vulnerability to distress during adolescence.

Practically, this scale can support:

1. School-based evaluation of puberty education programs, moving beyond knowledge tests to capture changes in attitudes, emotional readiness, and social meaning.
2. Targeted preventive interventions, especially where children exhibit negative affective evaluation interpretations towards pubertal changes.
3. Culturally informed programming, given the Indonesian context where puberty-related communication may be limited, and school may serve as a primary structured source of guidance.

Importantly, the three-factor model provides a framework for intervention design:

- The physical factor supports accurate, age-appropriate biological education.
- The psychological factor supports emotional regulation, identity development, and coping skills.
- The social factor supports peer-norm discussion, stigma reduction, and social confidence around body-related changes.

### 4.5 Limitations and future directions

Despite its strengths, the study has limitations that should be interpreted alongside the statistical results.

1. *Self-report constraints in younger children*. Because data were collected through self-report, responses may be influenced by social desirability and comprehension differences. While high reliability suggests consistency, younger participants may still interpret some items differently depending on language exposure and maturity.
2. *Context specificity*. The sample was drawn from a specific geographic/cultural context within Indonesia, limiting generalizability. Future work should test the scale in diverse Indonesian regions to capture potential variation in norms, school curricula, and family communication patterns related to puberty.
3. *Predictive validity and developmental trajectory*. Future longitudinal research should test whether attitudes assessed at ages 9–12 predict subsequent outcomes such as anxiety, body image concerns, coping strategies, and adjustment during early to mid- adolescence. This is especially important given the study’s theoretical framing that early attitudes may shape later adaptation.
4. *Further refinement of Factor 1 and item functioning*. The initial AVE shortfall for Factor 1 (0.465) suggested that some indicators did not share sufficient variance with the construct. The study addressed this through model respecification (see below), but future research could also explore item wording, reading level, and cultural nuance to strengthen construct representation further.

### 4.6 Model refinement, gender inclusivity, and conclusion of the discussion

A key contribution of the study is that it does not treat validity as a one-time outcome but as an iterative process supported by evidence-based refinement.

#### 4.6.1 Respecification and improved convergent validity

Because Factor 1 initially fell below the AVE criterion, the model was respecified by removing items 21 and 23 from Factor 1 due to relatively low factor loadings. After this refinement, convergent validity improved across all factors: AVE = 0.502 (F1), 0.525 (F2), 0.554 (F3), satisfying the ≥ 0.50 guideline. Discriminant validity also remained strong after respecification (HTMT = 0.406 to 0.788), with a reduced highest association between F1 and F2 (HTMT = 0.788), indicating improved construct separation.

#### 4.6.2 Measurement invariance across gender (MGCFA)

Critically, the scale’s gender- inclusive design was supported through sequential invariance testing:

- *Configural invariance*: CFI = 0.943, TLI = 0.930, RMSEA = 0.069 (90% CI [0.052, 0.085]), SRMR = 0.058, indicating the same factor structure holds across males and females.
- *Metric invariance*: CFI = 0.940, TLI = 0.931, RMSEA = 0.069, SRMR = 0.068, indicating equivalent factor loading strength across gender.
- *Scalar invariance*: CFI = 0.932, TLI = 0.928, RMSEA = 0.070, SRMR = 0.071, indicating intercept equivalence and supporting meaningful comparison of latent means between genders.

These findings support the conclusion that the scale measures puberty readiness/attitudes comparably for boys and girls, an important advance given prior Indonesian research gaps.

#### 4.6.3 Nuanced finding: higher F1–F2 association among males

One cautionary detail emerged: HTMT between Factor 1 and Factor 2 in the male group reached 0.910 (slightly above the 0.85 conservative cutoff) during invariance stages, suggesting these constructs may be more tightly linked for boys than girls. This does not invalidate the model especially because overall invariance and global fit remain acceptable but it suggests that, for boys, social and physical interpretations may be more intertwined. This is a useful direction for future research: examining whether boys’ pubertal attitudes are less differentiated due to socialization patterns, differences in puberty communication, or item content resonance.

#### 4.6.4 Overall conclusion

In line with the issues articulated in the introduction, the study demonstrates that puberty readiness/attitudes among Indonesian children in late childhood are multidimensional, measurable, reliable, and psychometrically supported. The instrument provides a foundation for advancing research on psychological readiness for puberty and supports early, culturally informed interventions to promote healthier adolescent development trajectories.

## 5 Summary (integrated with statistical evidence)

Through a systematic process of item development, expert validation, pilot testing, and rigorous psychometric evaluation, this study produced a valid, reliable, culturally appropriate, age-specific instrument to assess puberty readiness among Indonesian children aged 9–12 years. The scale demonstrated strong empirical support: meritorious sampling adequacy (KMO = 0.832), suitability for factor analysis (Bartlett’s *χ*^2^ = 2670.474, *p* < 0.001), an optimal three- factor structure supported by parallel analysis and scree plot, substantial explained variance (53.0%), strong item loadings after refinement, excellent CFA fit (CFI = 0.976, TLI = 0.971, RMSEA = 0.040, SRMR = 0.040), and high reliability (overall *ω* = 0.920; *α* = 0.902).

Validity evidence was strengthened through convergent and discriminant validity testing (AVE and HTMT), iterative refinement via respecification (improving AVE for Factor 1 from 0.465 to 0.502), and robust gender-based measurement invariance (configural, metric, and scalar). Collectively, these results indicate that the instrument is suitable for both research and applied contexts, enabling early assessment and evaluation of puberty education interventions in Indonesia while addressing a key methodological gap in the existing literature.

## Data Availability

The raw data supporting the conclusions of this article will be made available by the authors, without undue reservation.
