# Supplementary material for: Development and psychometric validation of the affective evaluation towards pubertal changes scale for late childhood (9–12 years old) Indonesian children
Source: Front Psychol. 2026 May 29;17:1793400. doi: 10.3389/fpsyg.2026.1793400 (PMC13261505; doi:10.3389/fpsyg.2026.1793400)
Supplement: Supplementary file 1 [file Table_1.DOCX]

**Table 1**. Items Removal

| **No** | **Items** | **Factor** | **Keep** | **Remove** |  |  |
| --- | --- | --- | --- | --- | --- | --- |
|  | Puberty makes my height and weight increase rapidly |  |  | Removed after EFA |  |  |
|  | Puberty changes add to the shopping list |  |  | Removed after EFA |  |  |
|  | Puberty changes make my armpits smell and I can use deodorant | Physical (Factor 3) | 🗸 |  |  |  |
|  | Puberty makes my body appearance and my friends’ bodies different from one another |  |  | Removed after EFA |  |  |
|  | Puberty makes me spend more time taking care of my body | \ |  | Removed after EFA |  |  |
|  | Puberty makes hair grow on my arms, legs, armpits, and genital area and I can shave it |  |  | Removed after EFA |  |  |
|  | Exercising helps in dealing with puberty |  |  | Removed after EFA |  |  |
|  | Puberty makes me easily hungry and I can eat more |  |  | Removed after EFA |  |  |
|  | Puberty makes my brain more developed |  |  | Removed after EFA |  |  |
|  | Puberty makes my face oily and I can take care of it | Physical (Factor 3) | 🗸 |  |  |  |
|  | Puberty makes me take better care of genital hygiene |  |  | Removed after EFA |  |  |
|  | Puberty makes my face, back, and chest get acne, and I can use acne cream | Physical (Factor 3) | 🗸 |  |  |  |
|  | Puberty makes me pay more attention to my appearance |  |  | Removed after EFA |  |  |
|  | Puberty makes me experience various new emotions more often |  |  | Removed after EFA |  |  |
|  | Puberty changes do not happen at the same time for everyone |  |  | Removed after EFA |  |  |
|  | Puberty means I am becoming a teenager and starting to be considered an adult | Psychological (Facror 2) | 🗸 |  |  |  |
|  | Puberty makes me explore who I am and what I like | Social (Factor 1) | 🗸 |  |  |  |
|  | Puberty makes me feel more mature | Psychological (Factor 2) | 🗸 |  |  |  |
|  | Puberty makes me more independent and responsible for myself | Psychological (Factor 2) | 🗸 |  |  |  |
|  | After puberty, I am no longer seen as a child | Psychological (Factor 2) | 🗸 |  |  |  |
|  | I feel more confident that I can do many things well after puberty |  |  | Removed after CFA modification |  |  |
|  | Puberty helps me know myself better | Social (Factor 1) | 🗸 |  |  |  |
|  | Puberty helps me discover the things that make me unique |  |  | Removed after CFA modification |  |  |
|  | After puberty, I can learn and understand more complex things |  |  | Removed after EFA |  |  |
|  | Puberty makes me do many fun activities with friends | Social (Factor 1) | 🗸 |  |  |  |
|  | Puberty makes friends more important than before | Social (Factor 1) | 🗸 |  |  |  |
|  | Puberty makes me more caring toward others | Social (Factor 1) | 🗸 |  |  |  |
|  | Puberty makes me attracted to the opposite sex |  |  | Removed after EFA |  |  |
|  | Puberty helps me make more friends | Social (Factor 1) | 🗸 |  |  |  |
|  | Puberty makes me want to be liked by others |  |  | Removed after EFA |  |  |
|  | Puberty makes me want to look attractive | Social (Factor 1) | 🗸 |  |  |  |
