# Supplementary material for: Development and psychometric validation of the affective evaluation towards pubertal changes scale for late childhood (9–12 years old) Indonesian children
Source: Front Psychol. 2026 May 29;17:1793400. doi: 10.3389/fpsyg.2026.1793400 (PMC13261505; doi:10.3389/fpsyg.2026.1793400)
Supplement: Supplementary file 2 [file Table_2.DOCX]

**Table 2**. Final Set of Questions Included in the Questionnaire

| **No.** | **Items** | **Factor** | **Answer** |  |  |
| --- | --- | --- | --- | --- | --- |
|  | Puberty changes make my armpits smell and I can use deodorant | Physical | 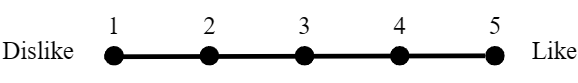 |  |  |
|  | Puberty makes my face oily and I can take care of it | Physical | 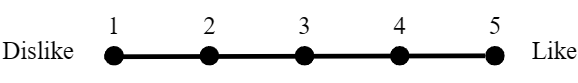 |  |  |
|  | Puberty makes my face, back, and chest get acne, and I can use acne cream | Physical | 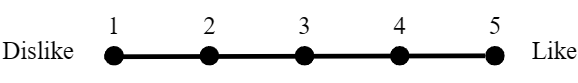 |  |  |
|  | Puberty means I am becoming a teenager and starting to be considered an adult | Psychological | 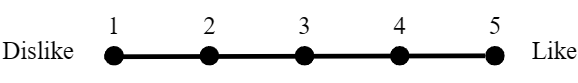 |  |  |
|  | Puberty makes me explore who I am and what I like | Psychological | 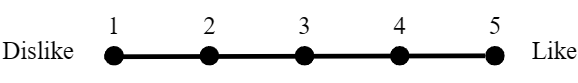 |  |  |
|  | Puberty makes me feel more mature | Psychological | 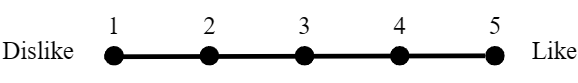 |  |  |
|  | Puberty makes me more independent and responsible for myself | Psychological | 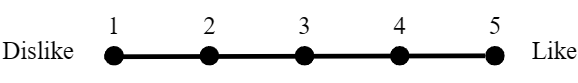 |  |  |
|  | After puberty, I am no longer seen as a child | Psychological | 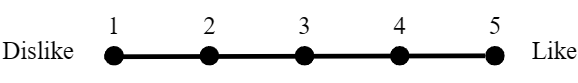 |  |  |
|  | Puberty helps me know myself better | Psychological | 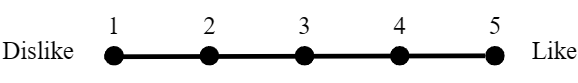 |  |  |
|  | Puberty makes me do many fun activities with friends | Social | 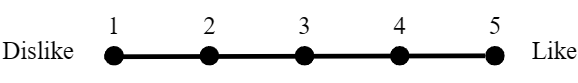 |  |  |
|  | Puberty makes friends more important than before | Social | 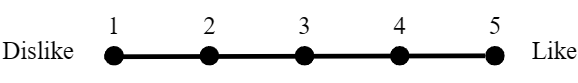 |  |  |
|  | Puberty makes me more caring toward others | Social | 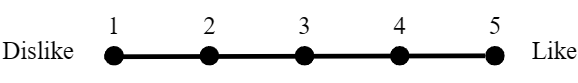 |  |  |
|  | Puberty helps me make more friends | Social | 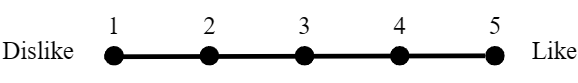 |  |  |
|  | Puberty makes me want to look attractive | Social | 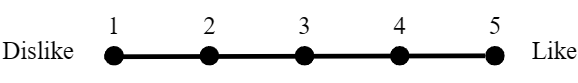 |  |  |
